# An Unusual Case of Pylephlebitis With the Involvement of Portal and Superior Mesenteric Veins

**DOI:** 10.7759/cureus.87679

**Published:** 2025-07-10

**Authors:** Camila Santos, Angel Vallejo, Jose Del Pino, Rolnel Manzano, Alex Villalta

**Affiliations:** 1 Department of Critical Care Medicine, Monte Sinaí General Hospital, Guayaquil, ECU; 2 Department of Pediatric Diagnostic Radiology, Roberto Gilbert Elizalde Hospital, Guayaquil, ECU

**Keywords:** anticoagulants, computed tomography scan, portal vein thrombosis, pylephlebitis, severe sepsis

## Abstract

Pylephlebitis is a low-incidence condition worldwide, more commonly affecting the adult population. Its clinical presentation is nonspecific, thus requiring a detailed clinical history and confirmation through imaging studies (contrast-enhanced computed tomography). The literature recommends antibiotic treatment for at least six weeks and the use of anticoagulants. When diagnosed and treated early, the prognosis is generally favorable. This report presents a case of pylephlebitis with clinical symptoms of fever, vomiting, and diarrhea. The patient was diagnosed with pylephlebitis involving the right and left portal veins and the superior mesenteric vein. She showed clinical improvement following treatment with broad-spectrum antibiotics and anticoagulants.

## Introduction

Pylephlebitis is defined as septic thrombophlebitis of the portal vein or of regions drained by the portal venous circulation. It is a rare condition with an incidence ranging from 0.37 to 2.7 cases per 100,000 person-years [1,2]. The most common etiology is intra-abdominal infections, with *Bacteroides* spp., *Escherichia coli*, and *Streptococcus* spp. being the most frequent pathogens [3]. Other risk factors for the development of pylephlebitis include hereditary prothrombotic disorders [4].

The clinical picture is characterized by abdominal pain, nausea, vomiting, and fever, making imaging studies essential for diagnosis [5]. Doppler ultrasound of the splenoportal axis can be useful for identifying thrombi, portal vein ectasia, hepatosplenomegaly, and ascites. However, contrast-enhanced computed tomography is the diagnostic method of choice, as it may show hypodense vascular thrombi and gas in the portal system [6,7]. 

The mainstay of treatment is a combination of anticoagulation and antibiotics. Surgical intervention is usually reserved for cases unresponsive to medical management or those requiring drainage of an infectious focus or abscess. Complications occur in approximately 20%-50% of the reported cases, including chronic portal vein thrombosis, mesenteric vessel infarction, portal hypertension, and hepatic abscesses. The reported mortality ranges from 11% to 55%, most commonly due to septic shock [7,8].

## Case presentation

We report the case of a 19-year-old female patient with a history of spontaneous abortion nine days prior to admission. She presented to the emergency department with a one-week history of unquantified fever, watery stools, and vomiting. On admission, her vital signs were: blood pressure, 140/70 mmHg (mean arterial pressure (MAP): 93 mmHg); heart rate, 74 bpm; respiratory rate, 18 breaths per minute; oxygen saturation, 97%; and temperature, 37.7°C. Physical examination revealed a soft, depressible, and tender abdomen in the epigastric region, left hypochondrium, and flank. Percussion was tympanic in all quadrants, bowel sounds were present, and McBurney, Blumberg, Rovsing, and Murphy signs were negative.

Initial workup included blood cultures, urine culture, stool culture, and laboratory tests, which showed normocytic normochromic anemia, leukocytosis, neutrophilia, and thrombocytopenia (Table 1). Urinalysis revealed a pH of 6, specific gravity of 1010, and leukocytes of 10/µL. Stool cytology showed 60% polymorphonuclear and 40% mononuclear cells. With a presumptive diagnosis of abdominal sepsis, empirical antibiotic treatment with piperacillin/tazobactam was initiated for eight days. The patient requested discharge.

**Table 1:** Laboratory Values on the first day of admission and re admission (8 days after) AST, aspartate aminotransferase; ALT, alanine aminotransferase; GGT, gamma-glutamyl transferase; LDH, lactate dehydrogenase; CRP, C-reactive protein.

Laboratory parameters	First day of admission	Re-admission (8 days after)	Normal Values
Hemoglobin	10.6 g/dl	9.5 g/dl	14-18 g/dl
White Blood Cells	21.0 uL	11.84 uL	4.00-10.00 uL
Neutrophils	55.5%	88.9%	50.00%-70.00%
Platelets	122.0 10³/ul	124.00 10³/ul	150.00-450.00 10³/ul
Total Bilirubin	0.96 mg/dl	2.79 mg/dl	0.30-1.10 mg/dl
Direct Bilirubin	0.75 mg/dl	2.14 mg/dl	0.0-0.30 mg/dl
Indirect Bilirubin	0.21 mg/dl	0.65 mg/dl	0.2-0.7 mg/dl
AST	177.1	38.2	0.0-35.0 U/L
ALT	168.0	40.4	0.0-32.0 U/L
GGT	303.9	384.6	8-35 U/L
Alkaline Phosphatase	210.0	156.0	<270 U/L
LDH	131.0	145.0	145-453 U/L
CRP	102.1	243.6	<10 mg/dL

Eight days later, she was readmitted with persistent symptoms: diarrhea and unquantified bilious vomiting. On re-admission, vital signs showed tachycardia, tachypnea, oxygen saturation 96%, hypotension, and fever. Physical exam revealed scleral icterus and epigastric and right hypochondrial tenderness. She was admitted to the intensive care unit.

Repeat cultures (blood, urine, stool) were negative. New lab results showed leukocytes, neutrophilia, anemia, and hyperbilirubinemia, with a predominance of direct, elevated liver enzymes and CRP (Table 1). Due to suspected abdominal sepsis and incomplete prior treatment, empirical antibiotic therapy with piperacillin/tazobactam was restarted.

**Figure 1:**
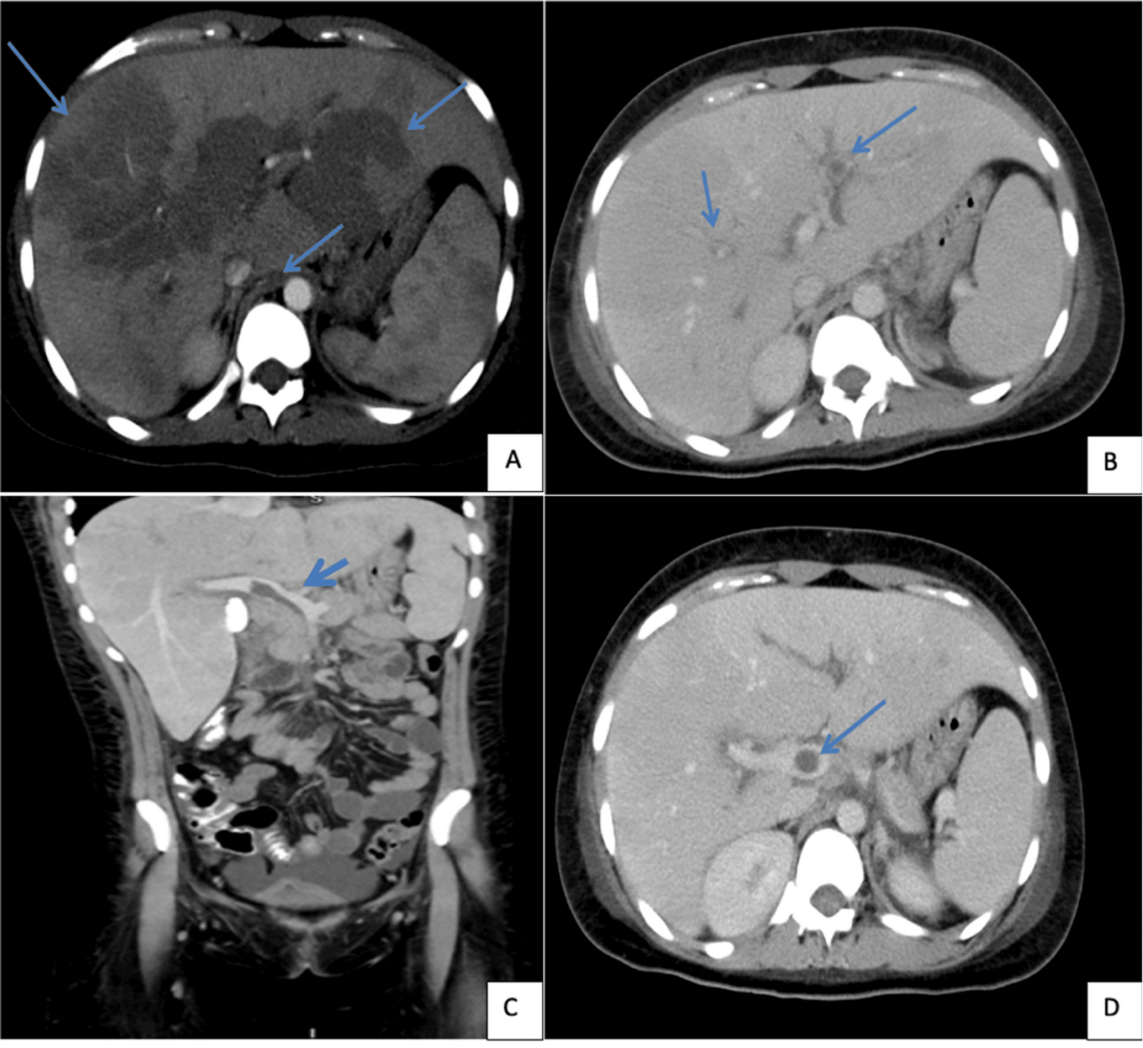
Contrast-enhanced abdominal CT arterial and venous phase Contrast CT scan of the abdomen showed lobulated liver with heterogeneous parenchymal attenuation, mainly periportal (left portal vein and anterior branch of right portal vein) (A, B). The CT also revealed hypodense content compatible with thrombus in the superior mesenteric vein before its confluence with the splenic vein, extending to the portal vein up to the hepatic hilum before bifurcating into right and left branches. A similar thrombus was found in the left portal vein branch (B,C,D)

Cholestatic syndrome and suspected cholangitis prompted an endoscopic retrograde cholangiopancreatography (ERCP) three days after admission, showing normal-caliber intrahepatic and extrahepatic bile ducts. With no infectious source identified, a contrast-enhanced abdominal CT was ordered. In the arterial phase, the CT showed a lobulated liver with heterogeneous parenchymal attenuation due to transient attenuation differences, mainly periportal (left portal vein and anterior branch of right portal vein), becoming isodense in the portal venous phase. There were no focal lesions or biliary dilation. The CT also revealed hypodense content compatible with thrombus in the superior mesenteric vein prior to its confluence with the splenic vein, extending to the portal vein up to the hepatic hilum before bifurcating into right and left branches. A similar thrombus was found in the left portal vein branch, associated with periportal edema (Figure 1).

A diagnosis of partial thrombosis of the right portal vein, superior mesenteric vein, and pylephlebitis was made. Subcutaneous enoxaparin every 12 hours was initiated, and antibiotic coverage was escalated to meropenem due to the risk of multidrug resistance.

Following anticoagulation and antibiotic escalation, the patient showed clinical improvement and was transferred to the internal medicine ward. She completed a 21-day course of meropenem and was discharged with oral dabigatran.

## Discussion

This case highlights pylephlebitis involving the right portal vein and superior mesenteric vein, a rare global condition that requires prompt and accurate diagnosis for immediate treatment initiation.

Pylephlebitis has a global incidence ranging from 0.37 to 2.7 cases per 100,000 person-years [1,2], with only 220 cases reported since 1975 [2]. It primarily affects adults; in a 2022 systematic review by Jevtic et al. [9] involving 103 cases, only 6.8% occurred in patients under the age of 18. However, Louro et al. (2021) [10-12] described a case in a 13-year-old boy who developed pylephlebitis without an identifiable primary source of infection. In a more recent 2023 systematic review by Fusaro et al., involving 220 patients with pylephlebitis, the most common symptoms were fever (75.5%) and abdominal pain (66.4%), while vomiting (25.5%), diarrhea (17.3%), and jaundice (12.7%) were less frequently reported [2]. These cases highlight the variability in clinical presentation across different age groups and the often nonspecific nature of the disease. In the present case, the patient just exhibited diarrhea, fever, jaundice, and vomiting.

Given its nonspecific presentation as abdominal pain, nausea, vomiting, and fever, detailed anamnesis and imaging are essential for accurate diagnosis. Common risk factors include prothrombotic states (e.g., Factor V Leiden mutation, prothrombin gene mutation, deficiencies in protein C/S and antithrombin III), essential thrombocythemia, paroxysmal nocturnal hemoglobinuria, pregnancy, and oral contraceptive use [4]. Our patient had a history of spontaneous abortion nine days prior, though its etiology was undetermined. 

The diagnosis of pylephlebitis can be challenging due to the absence of bacteremia in some cases. In a systematic review published in 2022 by Jevtic et al., which included 101 articles and a total of 103 patients diagnosed with pylephlebitis, 64 patients (62.1%) had positive blood cultures, while 21 cases (20.4%) had negative results. The most frequently isolated organisms were *E. coli* (20.4%), *Bacteroides* spp. (12.6%), *Streptococcus* spp. (11.7%), and *Fusobacterium* spp. (9.7%) [9]. Similarly, a retrospective study published in 2019 by Naymagon et al. reported that among 67 patients diagnosed with pylephlebitis, only 42% had positive blood cultures. [13]. In our clinical case, blood cultures were negative, which aligns with the findings of the aforementioned review and highlights that the absence of bacteremia does not exclude the diagnosis of pylephlebitis. This underscores the importance of a comprehensive clinical approach and the pivotal role of imaging studies in establishing a timely diagnosis, even in the absence of microbiological confirmation.

The gold standard diagnostic method is contrast-enhanced abdominal CT performed in both arterial and venous phases, although thrombosis may not be apparent within the first 48 hours. Jevtic et al., in their review of 103 patients diagnosed with pylephlebitis, demonstrated that diagnosis was established using CT scans in 89.3% of cases, whereas ultrasound was useful for diagnosis in only 38.8% of the patients. Any part of the portal system can be affected, though intrahepatic branches are most commonly involved. A systematic review of 95 cases published in 2015 by Choudhry et al., demonstrated that the most common sites of thrombosis include the right portal vein (33%), the main portal vein (32%), the superior mesenteric vein (31%-42%), the left portal vein (24%), the splenic vein (12%-18%), and the inferior mesenteric vein (2%-8%) [9-12]. In this case, CT confirmed thrombosis of the portal and superior mesenteric veins.

Historically, pylephlebitis was associated with a mortality rate of 50% to 80% in the absence of antibiotic therapy. Sepsis has been found to be the cause of death in approximately 88.9%. However, with the advent of early antibiotic treatment and the addition of anticoagulation, mortality has significantly decreased to approximately 11%-32% [3]. Nevertheless, Choudhry et al. reported a mortality rate of 11% in a cohort of 95 patients diagnosed with pylephlebitis who received antibiotic therapy, highlighting the potentially severe prognosis of this condition even with appropriate medical management. First-line treatment typically includes broad-spectrum antibiotics targeting Gram-negative aerobes and anaerobes, with therapy generally recommended for a minimum duration of six weeks [10-12]. Broad-spectrum regimens should be tailored based on culture results; however, Fusaro et al. reported that among 220 patients, the most commonly used therapy was a combination of metronidazole with either ceftriaxone, cefotaxime, ciprofloxacin, or levofloxacin. Monotherapy with a beta-lactamase inhibitor, such as piperacillin-tazobactam or ampicillin-sulbactam, was also frequently employed. Finally, carbapenem-based therapy included one of the following agents: imipenem, meropenem, or ertapenem [2]. In the present case, clinical improvement was observed following the enhancement of antimicrobial therapy to a carbapenem regimen.

Choudhry et al. (2016) [12] reported anticoagulant use in 82% of pylephlebitis cases, reflecting a notable increase compared to earlier studies where usage ranged from 35% to 70%. Anticoagulation is now widely recommended as a key component of management, aiming to limit thrombus propagation and promote its resolution. Supporting this, a retrospective study by Naymagon et al. (2020) [13] involving 67 patients with confirmed pylephlebitis demonstrated that anticoagulated patients had significantly higher rates of portal vein thrombosis resolution compared to non-anticoagulated patients (58% vs. 21%, p=0.0201), without an associated increase in major bleeding events. In 70.14% of the cases, the initial anticoagulant administered was intravenous heparin, which was subsequently transitioned to an oral or subcutaneous anticoagulant. The most commonly used agents included enoxaparin (41.30%), warfarin (36.95%), rivaroxaban (15.21%), apixaban (4.34%), and dabigatran (2.17%). Notably, anticoagulation was associated with improved outcomes, as only 6% of the patients who received anticoagulation died, compared to 20% mortality among those who did not receive anticoagulation. Furthermore, in a systematic review by Jevtic et al., the most commonly administered agents were heparin (28.2%), low-molecular-weight heparin (23.3%), warfarin (23.3%), and factor Xa inhibitors (11.7%) [9-13]. These findings underscore the importance of incorporating anticoagulation into therapeutic protocols for pylephlebitis to improve clinical outcomes. The patient in this case report was initially treated with subcutaneous enoxaparin and was discharged on oral anticoagulation with dabigatran.

Complications in pylephlebitis are common, particularly in patients who do not receive timely treatment. Thrombosis may persist or evolve into cavernous transformation of the portal vein. Additionally, the primary complications often result from hematogenous spread of the pyogenic portal infection, leading to metastatic abscess formation. Pyogenic liver abscesses occur in up to 37% of the cases, while other reported sites of metastatic abscesses include the brain and lungs [14]. In the present case, early and appropriate therapy was instrumental in preventing the development of such complications.

## Conclusions

Pylephlebitis of the portal vein and superior mesenteric vein is a condition with a low global incidence. However, it should be considered in patients presenting with fever, cholestatic syndrome, and prothrombotic risk factors. Early diagnosis and treatment can prevent major complications and are associated with a favorable prognosis. This case demonstrates favorable clinical evolution following early targeted therapy, emphasizing the importance of early diagnosis. Nonetheless, diagnosis remains challenging in the early stages due to nonspecific symptoms and low clinical suspicion.
